# Hippocampal sclerosis in women with temporal lobe epilepsy: seizure and pregnancy outcomes

**DOI:** 10.1186/s42494-024-00166-3

**Published:** 2024-09-23

**Authors:** Yujie Chen, Nanya Hao, Weixi Xiong, Hesheng Zhang, Enhui Zhang, Zhujing Ou, Lei Chen, Xintong Wu, Dong Zhou

**Affiliations:** Department of Neurology, West China Hospital of Sichuan University, No.37 Guoxue Alley, Wuhou District, Chengdu, 610041 Sichuan China

**Keywords:** Temporal lobe epilepsy, Hippocampal sclerosis, Pregnancy outcomes, Seizure management, Epilepsy in women, Retrospective studies, Anti-seizure medications

## Abstract

**Background:**

Temporal lobe epilepsy with hippocampal sclerosis (TLE-HS) is typically resistant to pharmacological interventions; however, achieving seizure freedom is possible through surgery. Our objective was to focus on the pregnancy and seizure outcomes during pregnancy of women with TLE-HS, and aim to identify predictors of seizure control.

**Methods:**

The West China Registry of Pregnancy of Women with Epilepsy (WCPR_EPi) was a monocentric prospective cohort study of women with epilepsy (WWE). We screened women with TLE-HS in this database. Their clinical profile, anti-seizure medication (ASM) use, and pregnancy outcomes were extracted from the records of the registry (2010–2023).

**Results:**

Out of 2320 WWE followed up, 47 pregnancies in women with TLE-HS were identified and analyzed. Seizure exacerbation occurred in 40.4% of pregnancies, and seizure freedom was present in 34.0% of these during pregnancy. Factors associated with seizure exacerbation during pregnancy was ASM non-adherence (odds ratio [OR] =7.00, 95% confidence interval [CI] 1.43–34.07,* P*=0.016). The surgery group showed a significantly higher seizure freedom rate (OR = 6.87, 95% CI 1.02–46.23,* P*=0.016) and lower rate of induced labor (0.0% vs 26.5%,* P*=0.047) compared to the medically-treated group alone. Caesarean section was chosen in 77.1% of cases due to seizure concerns, with comparable in epilepsy-related (*n*=20) and obstetric causes (*n*=24). No major congenital malformations were reported.

**Conclusions:**

Surgical treatment before pregnancy appears to offer a higher chance of seizure freedom compared to medication alone. Most of women with TLE-HS can deliver healthy offspring regardless of suboptimal seizure control and unwarranted concerns.

## Background

Epilepsy affects approximately 1% of the global population and poses significant challenges for women of childbearing age who have with epilepsy [1]. Managing seizures in pregnant women with epilepsy (WWE) is crucial to reduce the risks of obstetric complications [2]. Achieving seizure control during pregnancy presents a challenging dilemma for women, as anti-seizure medications (ASMs) have been associated with an elevated risk of major congenital malformations (MCMs) and neurodevelopmental delays in offspring [3, 4].

Seizure control during pregnancy is particularly challenging for women with focal epilepsy compared to those with generalized epilepsy, making medication management more complex [5, 6]. WWE in existing studies during pregnancy were those with good seizure control before conception, and a significant number of women remaining seizure-free throughout pregnancy [7]. However, there is a noticeable gap in the literature regarding the management of uncontrolled patients who become pregnant, and specific seizure types are rarely discussed.

In our study, we specifically focused on women with temporal lobe epilepsy and hippocampal sclerosis (TLE-HS) for three key aspects: First, debates persist regarding whether pre-pregnancy surgery reduces the risk of seizures during pregnancy [8–10]. Second, despite the typical drug resistance associated with temporal lobe epilepsy, ongoing use of ASM is often necessary. Third, the risk of increased seizures in this population is not yet fully understood, and there is limited pregnancy-related data available from developing countries [11, 12].

The objectives of our study are to: i) characterize seizure and pregnancy outcomes in women with TLE-HS; ii) compare outcomes between women treated only with medication (WWE-MO) and those who underwent surgery (WWE-S); iii) identify predictors of increased seizure risk. Our findings may assist clinicians in managing this prevalent type of refractory epilepsy during pregnancy.

## Methods

### Data acquisition and patient cohort

Women with epilepsy seeking pregnancy-related advice at West China Hospital were informed about our study. They were prospectively registered in the West China Registry of Pregnancy of Women With Epilepsy (WCPR_EPi) from January 2010 to December 2023. The inclusion criteria for WWE were ages 18–45 years and a gestational age of ≤ 24 weeks. Exclusion criteria included WWE who refused to participate in the pregnancy registry.

A standardized case report form (CRF) and corresponding work manual were designed by a counselling group to collect data from patients at outpatient department and epilepsy clinic every three months untill the delivery or termination of pregnancy. A final follow-up was one year later. In cases where a patient missed a clinical visit, attempts were made to contact them via phone to gather necessary information. For this study, we conducted a retrospective analysis of data from the registry. Patients diagnosed with TLE-HS by the end of the first trimester of pregnancy were selected from the registry database. Exclusion criteria included loss of follow-up and presence of extra-temporal lesions.

### Diagnostic criteria for TLE-HS

The diagnosis of epilepsy follows the criteria set by the International League Against Epilepsy (ILAE) [13]. In our study, we screened women with TLE-HS using the following additional criteria [14–16]: i) presence of epigastric sensation, feelings of *déjà vu* or *jamais vu,* or olfactory aura; ii) experience of oral or manual automatism, dystonic posturing of one hand, staring lasting 0.5–2 minutes, unexpected fear, or notable autonomic signs; iii) detection of temporal lobe interictal epileptiform discharges and temporal and/or frontocentral and/or ictal discharge; iv) identification of hippocampal sclerosis (HS) based on magnetic resonance imaging (MRI) on a 3.0-Tesla system, including a reduced hippocampal volume on T1-weighted imaging, increased signal intensity on T2-weighted imaging, dilatation of the temporal horn, and blurring of the gray-white matter interface [17]. To qualify for inclusions, patients must meet either i) or ii), as well as both iii) and iv). The diagnosis was verified by two experienced neurologists (DZ and XW).

### Variables and outcomes

In this study, we collected data on various variables including demographics, medical conditions, folic acid supplementation, epilepsy type, seizure frequency, seizure types, drug therapy, and other potential risk factors. Patients' adherence to the prescribed daily medication dose was specifically assessed. All patients were asked to maintain a diary to document seizure occurrences and any changes in ASMs. The seizure outcomes investigated in this study included the percentage of women experiencing seizure exacerbation, achieving or maintaining seizure freedom throughout the pregnancy, and seizure occurrence during delivery or the postpartum week. Baseline seizure frequency was determined from the year before conception, and categorized as daily (≥2 times per day on most days), multiple per day (4–6 per week), weekly (1–3 per week), monthly (1–3 per month), quarterly(4–6 per year), yearly (1–3 time per year), or seizure-free in the preconceptional year, which served as the reference point [18]. Seizure exacerbation was defined as transitioning from a lower to a higher frequency category compared to the baseline during any trimester of pregnancy. Seizure types were categorized as focal to bilateral tonic-clonic seizure (FBTCS), focal impaired awareness seizure (FIAS), or focal onset aware, as previously defined [19].

Clinical records were reviewed to verify pregnancy outcomes, including the type of delivery, live birth or fetus loss (spontaneous abortions, intrauterine deaths, stillbirth), and birth weight below 2500 g or Apgar score < 7. Major malformations were defined as structural abnormalities with surgical, medical, or cosmetic importance, while minor malformations referred to relatively mild or less severe structural or anatomical abnormalities typically present at birth but has limited medical, functional, or cosmetic impact, such as benign cysts.

In the subsequent analysis, participants were further categorized into two groups: the WWE-S group, consisting of individuals who underwent surgery before their pregnancies, with or without medication usage during pregnancy, and the only medically-treated group (WWE-MO), consisting of patients exclusively treated with medication. We compared the two groups in terms of demographic data, epilepsy characteristics, epilepsy outcomes, as well as pregnancy and fetal outcomes.

### Statistical analysis

The statistical analysis was performed using SPSS 26 (IBM, Chicago, IL, USA) with a significance level set at two-sided *P* < 0.05. Continuous data were reported as mean (SD) if normally distributed (assessed using the Shapiro-Wilk test), or as median (interquartile range, IQR) if skewed. Categorical data were reported as counts and percentages. Differences in subgroups were further assessed for significance ascertained by Student’s *t*-test or Mann-Whitney U tests for quantitative variables. For categorical variables, the chi-squared test (assessed by evaluating contingency table cell counts *n*≥40 and theoretical expected frequencies E≥5) or Yates' continuation correction (*n*≥40 and 1≤E<5) or Fisher exact test (*n*<40 or E<1) was used. Pregnancy outcomes were presented with description data. Separate logistic regression models were employed to calculate the OR (odds ratio) for each outcome, examining the association between pre-conceptional surgery and ASM use with the likelihood of seizure exacerbation and seizure freedom. Variables with high collinearity (variance inflation factor >5) were excluded from the multivariate model.

## Results

From January 2009 to December 2023, a total of 2320 WWE were followed up at West China Hospital. Among them, 52 pregnancies were identified with TLE-HS. After screening, exclusions were made for loss of follow-up (*n*=2) and extra-temporal lesions (*n*=3). Ultimately, 47 pregnancies were finally included in the analysis. The mean age of participants was 25.7 years, and the median duration of epilepsy was 9.0 years. None of the participants had solely focal onset aware seizures, so the analysis focused on FBTCS and FIAS.

### The pregnancies studied

**Fig. 1:**
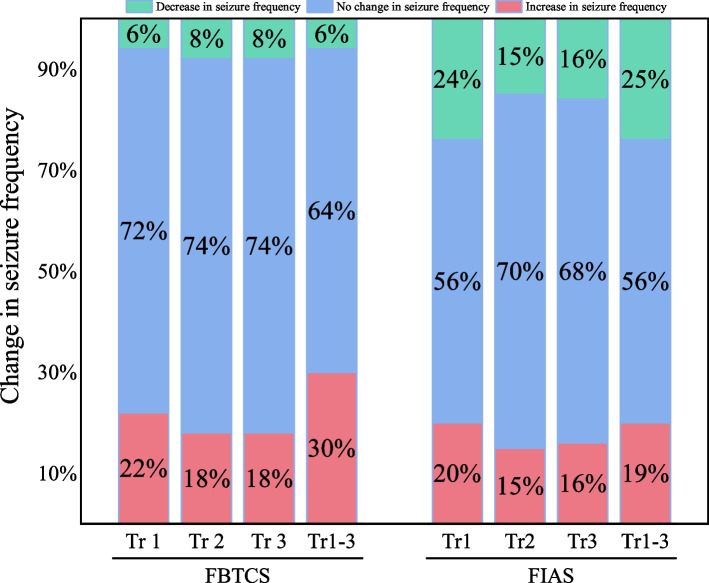
Trimester change in seizure frequency for two seizure types. Abbreviation:Tr: trimester; FBTCS: focal to bilateral tonic-clonic seizure; FIAS: focal impaired awareness seizure

Demographic and clinical data for the enrolled cases are presented in Table 1. Among the participants, 9 women (19.1%) had remained seizure-free in the year before conception. Of those who were seizure-free before conception, 8 (88.9%) remained seizure-free throughout pregnancy. Approximately two-thirds of women had no FBTCS in the preconception year, while a few (8, 17.0%) experienced frequent FIAS with more than one episode per week. During pregnancy, 19 (40.4%) pregnancies experienced seizure exacerbation, with 14 pregnancies (29.8%) showing an increase in FBTCS and 9 (19.1%) having an increase in FIAS. No specific trimesters were identified for seizure exacerbation. Changes in seizure frequency are summarized in Fig. 1.

**Table 1:** Clinical characteristics of patients included in the study (*n*=47)

Variable	Mean(±SD)/Value(rate)
Age at pregnancy mean (SD)	25.7 (3.0)
Educational years (*n,* %)	
< 9 year	17 (36.1%)
9–12 year	11 (23.4%)
> 12 year	19 (40.4%)
Seizure freedom in the preconceptional year	9 (19.1%)
Duration of epilepsy in years (median, IQR)	9.0 (7.0-12.0)
FBTCS frequency in the periconceptional year	
None	31 (66.0%)
Yearly	8 (17.0%)
Quarterly	4 (8.4%)
Monthly	3 (6.4%)
Weekly	1 (2.1%)
FIAS frequency in the periconceptional year (*n,* %)	
None	15 (31.9%)
Yearly	4 (8.5%)
Quarterly	3 (6.4%)
Monthly	17 (36.2%)
Weekly	4 (8.5%)
Daily	1 (2.1%)
Multiple times per day	3 (6.4%)
ASM administration before pregnancy (*n,* %)	
LEV	14 (29.8%)
LTG	12 (25.5%)
CBZ	9 (19.1%)
TPM	8 (17.0%)
VPA	8 (17.0%)
Seizure exacerbation during pregnancy (*n*, %)	19 (40.4%)
Seizure freedom during pregnancy (*n*, %)	16 (34.0%)
Seizure occurrence in the postpartum week (*n*, %)^b^	7 (20.0%)
Number of ASMs during pregnancy (*n,* %)	
None	11 (23.4%)
One	21 (41.7%)
More than one	15 (31.9%)
ASMs administration during pregnancy (*n,* %)	
LEV	18 (38.3%)
LTG	13 (27.7%)
CBZ	9 (19.1%)
TPM	4 (8.5%)
VPA	3 (6.4%)
ASM adherence during pregnancy (*n,* %)^b^	29 (67.4%)^a^
ASMs adjustment (*n,* %)	14 (32.6%)^a^
Fetal loss (*n,* %)	
Spontaneous abortion	3 (6.4%)
Induced abortion	9 (19.1%)
Cesarean section (*n,* %)^a^	27 (77.1%)^b^
Birth weight < 2.5kg, (*n,* %)^a^	3 (8.6%)^b^
Apgar scores (< 7) (*n,* %)	0 (0.0%)
Major congenital malformations (*n,* %)	0 (0.0%)

*Abbrevations: SD* standard deviation, *FBTCS* focal to bilateral tonic-clonic seizure, *FIAS* focal impaired awareness seizures, *ASM* anti-seizure medication, *LEV* levetiracetam, *LTG* lamotrigine, *CBZ* carbamazepine, *TPM* topiramate, *VPA* valproic acid, *IQR* Interquartile range

a. Cases involving 4 pregnancies who had successfully discontinued ASMs were excluded. b. Cases involving 12 abortions were excluded

Data on fetal losses were recorded in 12 pregnancies (25.5%), with 9 of these (75%) identified as induced abortions. The reasons for induced abortions were categorized as concerns about epilepsy inheritance (*n*=5), concerns about the influence of ASMs or seizures (*n*=3) and fetal death without autopsy (*n*=1). Among the nine pregnancies resulting in induced abortions, seven had no FBTCS in the year before abortion, while the remaining two had FBTCS. The ASM use and seizure control of the WWE with unexpected fetal losses was showed in Table 2.

**Table 2:** ASM use of 3 patients (4 pregnancies) with unexpected fetal losses before and during pregnancy

	Fetal outcomes	ASM use before pregnancy	Time	ASM change during pregnancy	Seizure frequency before pregnancy	Frequency change during pregnancy	Folic acid intake
Patient 1	fetal death	LEV 0.5g bid	Week 13	stopped and resumed later	FIAS: yearly; FBTCS: yearly	increase (six times per month)	800μg/day qd for the first three months of pregnancy
Patient 2	spontaneous abortion	VPA 0.4g bid; CBZ 0.2g bid	Week 4	no change	FIAS: daily	no change	no
Patient 2	spontaneous abortion	VPA 0.4g bid; CBZ 0.2g bid; TPM 50mg bid;	Week 8	no change	seizure-free for three years after surgery	no change	no
Patient 3	spontaneous abortion	LTG 50mg bid; LEV 0.5g bid	Week 6	stopped and resumed later	FIAS: monthly; FBTCS: seizure free for more than a year	no change	400μg/day qd for the first three months before pregnancy

*Abbreviation: FBTCS* Focal to bilateral tonic-clonic seizure, *FIAS* Focal impaired awareness seizures, *ASM* Anti-seizure medication, *LEV* Levetiracetam, *LTG* Lamotrigine, *CBZ* Carbamazepine, *TPM* Topiramate, *VPA* Valproic acid

None of the live offspring from the pregnancies in women with TLE-HS had major congenital malformations. Only one offspring had an umbilical hernia, which resolved within three months after birth. Among the pregnancies, 77.1% (*n*=27) opted for caesarean section delivery. The choice of caesarean section was made due to various reasons, including epilepsy-related concerns (*n*=20, 8 of which were advised by obstetricians) and obstetrician-related concerns (*n*=24), including prolonged labor (*n*=4), previous cesarean section delivery history (*n*=4), cephalopelvic disproportion (*n*=4) circular of the umbilical cord (*n*=3), premature rupture of membrane (*n*=3), and preeclampsia (*n*=2). Among the pregnancies with seizure concerns (*n*=18), 14 individuals experienced an increase in seizures during pregnancy, and 11 of them had an increase specifically in the third trimester. In one case, the pregnancy was complicated by epileptic seizures during delivery, during which the woman experienced exacerbation of FBTCS throughout pregnancy. The Apgar scores for all live births were between 7 and 10. Additionally, three infants were born with low birth weights (2050g, 2000g, and 2000g, the latter two were twins), but their weight normalized within one year of follow-up. The WWE of these infants experienced monthly FIAS.

### ASMs administration and seizure control

Roughly one-third of pregnancies were exposed to polytherapy during pregnancy, with 86.7% of these cases involving the combination of two ASMs. Levetiracetam emerged as the most frequently administered ASM, accounting for 38.3% of usage, followed by lamotrigine at 27.7%.

**Fig. 2:**
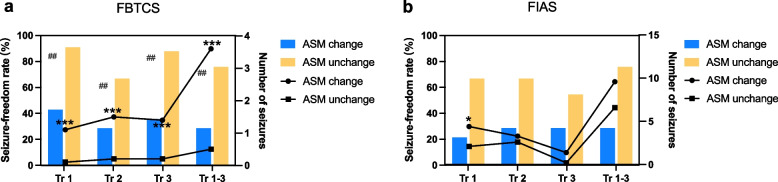
Seizure control in each trimesters between WWE with and without ASMs change by seizure types. **a** The occurrence and numbers of FBTCS seizures are compared between the two groups (with or without ASMs change) in each trimester of WWE **b** The occurrence and numbers of FIAS seizures are compared between the two groups (with or without ASMs change) in each trimester of WWE. Abrrevations: Tr: trimester; FBTCS: focal to bilateral tonic-clonic seizure; FIAS: focal impaired awareness seizure. The number of seizures was expressed as mean and the comparisons between the two groups were performed using the Wilcoxon rank sum test, as the data were not normally distributed. χ^2^ test or Fisher's exact test was performed to compare the seizure-freedom rate. For number of seizures : *P < 0.05, **P < 0.01 and ***P < 0.001; For seizure frequency rate: ^#^*P* < 0.05, ^##^*P* < 0.01

Among the 47 pregnant women observed, 70.2% (*n*=33) maintained a consistent ASM dosage throughout their trimesters. Notably, pregnancies with unchanged ASM dosage exhibited a higher prevalence of fully-controlled FBTCS and fewer seizure occurrences compared to those with ASM adjustments. FIAS were relatively unaffected by ASM adjustments (Fig. 2). ASM adjustments, accounting for 32.6% of pregnancies (*n*=14), primarily involved withdrawal of ASMs (*n*=7) or dose increase/addition (*n*=7) of newer medications such as levetiracetam, oxcarbazepine, and lamotrigine. Six out of seven patients who underwent dose increase/addition of medications experienced frequent FBTCS (≥5 times during pregnancy). Preemptive dose increase of oxcarbazepine was done in the remaining case, with seizure freedom throughout pregnancy and monthly FIAS before conception. Six out of seven women who withdrew ASMs during early gestation experienced increased FBTCS. Among these cases, five were related to valproate acid. However, all three women who restarted ASMs regained seizure stability. In fact, six individuals discontinued medication prior to pregnancy despite experiencing epileptic seizures. As a result, thirteen individuals (30.2%) were non-compliant with their medication regimen.

Interestingly, two women who previously had weekly FIAS became seizure-free during pregnancy despite withdrawing ASMs in the first trimester. Both individuals noted an increased frequency of seizures during menstruation. Furthermore, another woman experienced the onset of epilepsy for the first time during her pregnancy.

### WWE-S vs WWE-MO

There were no significant differences in demographic characteristics, age, and seizure duration between WWE-S and WWE-MO groups (Table. 3). The WWE-S group had an average time of 10.1 years from epilepsy diagnosis to surgery. All women in the WWE-S group underwent temporal resection. The median time to achieve pregnancy after surgery was 4.5 years, ranging from 2.5 to 7 years (IQR). Five women experienced recurrent seizures after surgery before pregnancy (38.5%), while the majority of individuals in the WWE-MO group did not achieve seizure freedom before pregnancy (97.1%, *P*<0.001). Overall, the rate of seizure exacerbation during pregnancy was lower in the WWE-S group compared to the WWE-MO group (15.4% in WWE-S vs 50.0% in WWE-MO, *P*=0.031). As expected, the WWE-S group exhibited a significantly higher rate of seizure freedom rate compared to the WWE-MO group (76.9% vs 17.6%, *P*<0.001).

**Table 3:** The comparisons of women with epilepsy surgically-treated (WWE-S) (*n*=13) versus medication-treated only (WWE-MO) (*n*=34)

Variable	WWE-MO (*n*=34)	WWE-S (*n*=13)	*P*-value
Age at pregnancy mean (SD)	25.4 (3.0)	26.3 (3.1)	0.92
Educational years >12 (*n*, %)	15 (44.1%)	4 (30.8%)	0.82
Duration of epilepsy (years, median,IQR)	9.0 (6.0–10.0)	10.0 (7.5–14.0)	0.26
Seizure freedom in the pre-conceptional years (*n*,%)	1 (2.9%)	8 (61.5%)	0.001*
FBTCS frequency (none) in the preconceptional year (*n*,%)	22 (64.7%)	11 (84.6%)	0.030*
FIAS frequency (monthly) in the preconceptional year	15 (44.1%)	2 (15.4%)	0.003*
Levetiracetam use before pregnancy (*n*, %)	10 (29.4%)	4 (30.8%)	0.99
Seizure exacerbation during pregnancy (*n*, %)	17 (50.0%)	2 (15.4%)	0.031*
Seizure freedom during pregnancy (*n*, %)	6 (17.6%)	10 (76.9%)	<0.001*
Seizures in the postpartum week (*n*, %)	6 (17.6%)	1 (7.7%)	0.69
Polytherapy during pregnancy (*n*, %)	9 (26.5%)	6 (46.2%)	0.37
Levetiracetam use during pregnancy (*n*, %)	13 (38.2%)	5 (38.4%)	0.99
ASM adherence (*n*, %)	21 (61.8%)	8 (88.9%)^a^	0.30
Induced delivery (*n*, %)	9 (26.5%)	0 (0.0%)	0.047*
Cesarean section (*n*, %)	19 (55.9%)	8 (61.5%)	0.21

*Abbreviations: WWE-MO* Medication-only treated women with epilepsy, *WWE-S* Surgically treated women with epilepsy

^*^P-value

Among the pregnancies following surgery, four successfully withdrew from ASM before pregnancy. Polytherapy was observed in 46.2% (6/13) of women in the WWE-S group compared to 26.5% (9/34) in the WWE-MO group (*P*=0.37). There were no significant differences between the WWE-S and WWE-MO groups in terms of the choice and the number of ASMs. In our study, three women had surgery after the first pregnancy and experienced an uneventful second pregnancy. It's worth noting that none of the women in the WWE-S group chose induced abortions, while 26.5% of WWE-MO group chose induced abortions (*P*=0.047).

### Predictors of seizure control during pregnancy

Multivariate logistic regression analysis revealed a notable association between increased seizure frequency and non-adherence to ASM in WWE (odds ratio [OR]=7.00, 95% confidence interval [CI] 1.43–34.07, *P*=0.016). Additionally, our findings indicated a higher likelihood of achieving seizure freedom among women with prior surgery (OR=6.87, 95% CI 1.02–46.23, *P*=0.047), and among women who were seizure-free in the year preceding conception. No significant collinearity was observed among the variables considered (Table 4).

**Table 4:** Multivariate regression analysis considering seizure freedom and seizure exacerbation during pregnancy

Variable	Seizure freedom					Seizure exacerbation				
	Event	OR (95%CI)	*P*	VIF	*R*^*2*^	Event	OR(95%CI)	P	VIF	*R*^*2*^
Surgery before conception	10 (62.5%)	6.87 (1.02,46.23)	0.047	1.65	0.26	2 (10.5.%)	0.15 (0.01,1.67)	0.12	1.65	0.25
ASM non-adherence	2 (12.5%)	0.68 (0.09,4.83)	0.68	1.09		2 (10.55.3%)	7.00 (1.43,34.07)	0.016	1.09	
Pre-conceptional seizure freedom	9 (56.3%)	19.61 (1.74,221.00)	0.016	1.59		1 (5.3%)	0.32 (0.02,4.77)	0.41	1.60	

*Abbrevations: CI* confidence interval, *OR* odds ratio, *FBTCS* focal to bilateral tonic-clonic seizure, *FIAS* focal impaired awareness seizures, *ASM* Anti-seizure medication, *VIF* variance inflation factor

*R*^*2*^ values relate to Cox and Snell R-square

## Discussion

This study is the inaugural analysis of seizure control and pregnancy outcomes in women with HS, revealing that 40% of those with TLE-HS experienced an escalation in seizures compared to the preconception period, while only a third maintained a seizure-free status. Surgical intervention and adherence to treatment significantly improved seizure control. Additionally, three quarters of pregnancies opted for induced abortions, ASM withdrawal and cesarean section due to concerns related to epilepsy. However, despite the high rate of seizure exacerbation and the relatively low rate of maintaining seizure-free status, the majority of women delivered normal, healthy offspring in the one-year follow up.

Previous observational studies have reported that focal epilepsy, although typically more challenging to be controlled than generalized epilepsies [20], often presents a favorable pattern during pregnancy [21, 22]. The condition is marked by fluctuations in seizure frequency, but about 70% of cases remain stable and more than 60% achieved seizure freedom. For instance, Voinescu's study reported among about 47 pregnancies in subgroup set and most were temporal lobe epilepsies [23], only a modest increase (11.9%) in seizure frequency during pregnancy, with the majority maintaining seizure-free status. There are two reasons accounting for these gaps. First, the low rate of seizure freedom in the pre-conception period, particularly in women with refractory epilepsy, especially TLE-HS. It has been well-established that the occurrence of seizures before pregnancy is a critical predictor of seizures during pregnancy [24]. The intrinsic selection bias from the peculiar group is unavoidable when selecting the group with uncontrolled seizures, which was one of the intentions in this research. Additionally, nearly one-fifth of pregnancies in our cohort exhibited poor adherence to treatment, which is concerning as poor compliance is associated with FBTCS in women with TLE. It is essential to emphasize compliance with prescribed ASM therapy throughout pregnancy to avoid breakthrough seizures. Pre-pregnancy counseling and planned pregnancies are also crucial to reduce the rates of inappropriate valproate usage [25, 26].

Women with TLE often have unnecessary concerns the impact of epilepsy on pregnancy, leading them to discontinue medication, terminate pregnancies and undergo cesarean section. The high rate of cesarean section in our data (2009–2023) from Southwest China (79.4%) was found to be comparable to that (2006–2019) in Northern China (85.4%) [27], and notably, it was twice as high as the rate observed in developed countries [10, 28, 29]. Half of them choose cesarean section for epilepsy-related reasons, despite the infrequency of convulsive seizures in the preconception period. In fact, seizures during pregnancy are uncommon in our cohort (2.6%, 1 of 38), which is consistent with the EURAP study (20). Furthermore, similar to a Japanese cohort study [30], we found that the majority of WWE during pregnancy had favorable neonatal outcomes, in spite of frequent seizures during pregnancy and a lack of adherence to medication by the mothers. Therefore, it is crucial to emphasize the improvement of personal education and emotional support for WWE and neurologists also play a vital role in providing advice and guidance to them.

In our study, a majority of WWE-S achieved enhanced seizure control during pregnancy compared to those treated with ASMs only. HS is recognized as a primary surgical condition, and it can lead to superior seizure control, with a noteworthy subset of patients even managing to successfully discontinue ASMs. This highlights the significance of timely intervention to address this condition [31]. Approximately 70% of individuals with this disorder have the potential to achieve freedom from seizures within a span of 5 years after undergoing epilepsy surgery [32]. After epilepsy surgery, a smaller proportion of WWE-S patients used polytherapy during pregnancy yet achieved seizure freedom. This can be partly attributed to inadequate follow-up and adjustments in medication dosage. Some patients may opt to continue medication due to fear of relapse. Furthermore, in our epilepsy center, women are more likely to maintain pregnancy and pursue a second child after surgery, which aligns with China's Second-Child Policy.

Among Chinese pregnant WWE, levetiracetam is preferred over lamotrigine. This preference is driven by the desire to avoid allergic cutaneous reactions, especially considering the variable occurrences of HLA-B*1502 associated with such reactions in 10–15% of individuals in southern China [33].What’s more, lamotrigine levels tend to drop notably during pregnancy [34, 35]. In our cohort, women who underwent changes in ASMs were found to experience a higher frequency of seizures. One reason for this was that one third of them were non-compliant with treatment. During pregnancy, if seizures occurred without a trigger, medication dosage was carefully adjusted, and additional ASMs were cautiously introduced, particularly evident in cases of FBTCS. This approach aligns with previous studies indicating that frequent tonic-clonic seizures pose potential risks for developmental delay and cognitive impairment [3, 36]. Notably, proactive adjustment of ASM dosage during pregnancy by one patient led to seizure freedom. However, routine therapeutic drug monitoring was not feasible due to limited accessibility in our centers, and challenges in establishing regular clinics in remote areas. In addition, we were unable to compare ASM concentrations during pregnancy with pre-pregnancy concentrations in many women due to the fact that they often conceived while attending clinics, which could lead to misleading conclusions. While newer ASMs may necessitate drug level monitoring during pregnancy [37], a double-blind randomized trial involving 263 pregnant women found that monitoring drug levels for newer ASMs did not significantly improve epilepsy management compared to monitoring clinical features alone [38]. Future research should focus on guiding appropriate therapy adjustments for WWE during pregnancy.

Intriguingly, some women who had frequent seizures before pregnancy achieved seizure freedom without the use of ASMs during pregnancy, reporting a catamenial pattern. This phenomenon may be influenced by hormonal changes and other factors, such as random fluctuations. Notably, seizures linked to hormonal changes represent a distinctive feature of catamenial epilepsy. Catamenial epilepsy is characterized by heightened seizure frequency during specific menstrual cycle phases, is frequently observed in temporal lobe epilepsy [39]. Women with catamenial epilepsy are more likely to achieve seizure freedom and experience reduced seizure frequency throughout pregnancy [40]. However, it is important to confirm these observations prospectively through seizure diary and hormone level montoring [41].

The disease studied in our research is a most common type of drug-resistant epilepsy characterized by favorable surgical outcomes. However, it is important to note that the finding in favor of preconceptional surgery cannot be generalized to other types, such as MRI-negative epilepsy [42]. Moreover, as our study was conducted in the tertiary epilepsy center, the management of pregnant women with epilepsy by non-neurologists or non-epileptologists in other healthcare settings may not meet the standards. It is imperative to underscore those aspects such as seizure control and epilepsy management may demonstrate commonalities across various forms of drug-resistant epilepsy.

The present study has several limitations. Firstly, the sample size was limited, we didn’t identify any significant congenital malformations or irreversible abnormalities in the one-year follow-up period. The variability in seizure frequency among individuals highlights the need for a larger sample size to minimize the risk of Type II errors. Additionally, the regression analyses were constrained by the small sample size, limiting the ability to address potential confound. Furthermore, the precision of the fetal loss rate might also be influenced by the small sample size, hindering further exploration of the impact of ASM or seizure control on this outcome.

Diagnosing HS primarily relies on imaging, with an accuracy of only 70% [43]. It is possible that some cases of HS were overlooked in our cohort, which might impact the overall findings. The follow-up time in the postpartum period was relatively short, which may not have captured changes in seizure frequency after delivery.

## Conclusions

In conclusion, effective management of epilepsy in expectant WWE is crucial for the health of both the mother and the newborn. Our study indicates that women with TLE-HS may face an increased risk of seizure exacerbation during pregnancy. However, our findings suggest that this risk can be mitigated through the consistent use of ASMs and thoughtful consideration of surgery before pregnancy when appropriate.

## Data Availability

All data are available from the corresponding author upon reasonable request.

## Abbreviations

ASM: Anti-seizure medication
CBZ: Carbamazepine
FBTCS: Focal to bilateral tonic-clonic seizure
FIAS: Focal impaired awareness seizure
IQR: Interquartile range
LEV: Levetiracetam
LTG: Lamotrigine
MCM: Major congenital malformation
TLE-HS: Temporal lobe epilepsy with hippocampal sclerosis
TPM: Topiramate
Tr: trimester
VIF: Variance inflation factor
VPA: Valproic acid
WWE: Women with epilepsy
WWE-MO: Medication-only treated women with epilepsy
WWE-S: Surgically treated women with epilepsy
